# Association between Green Space Structure and the Prevalence of Asthma: A Case Study of Toronto †

**DOI:** 10.3390/ijerph18115852

**Published:** 2021-05-29

**Authors:** Yuping Dong, Helin Liu, Tianming Zheng

**Affiliations:** 1 School of Architecture and Urban Planning, Huazhong University of Science and Technology, Wuhan 430074, China; youkie@hust.edu.cn (Y.D.); tm1996@hust.edu.cn (T.Z.); 2 Center for Urban and Rural Planning Support Research, Huazhong University of Science and Technology, Wuhan 430074, China

**Keywords:** green space structure, prevalence of asthma, air pollution, tree diversity, pathways, Toronto

## Abstract

Asthma is a chronic inflammatory disease that can be caused by various factors, such as asthma-related genes, lifestyle, and air pollution, and it can result in adverse impacts on asthmatics’ mental health and quality of life. Hence, asthma issues have been widely studied, mainly from demographic, socioeconomic, and genetic perspectives. Although it is becoming increasingly clear that asthma is likely influenced by green spaces, the underlying mechanisms are still unclear and inconsistent. Moreover, green space influences the prevalence of asthma concurrently in multiple ways, but most existing studies have explored only one pathway or a partial pathway, rather than the multi-pathways. Compared to greenness (measured by Normalized Difference Vegetation Index, tree density, etc.), green space structure—which has the potential to impact the concentration of air pollution and microbial diversity—is still less investigated in studies on the influence of green space on asthma. Given this research gap, this research took Toronto, Canada, as a case study to explore the two pathways between green space structure and the prevalence of asthma based on controlling the related covariates. Using regression analysis, it was found that green space structure can protect those aged 0–19 years from a high risk of developing asthma, and this direct protective effect can be enhanced by high tree diversity. For adults, green space structure does not influence the prevalence of asthma unless moderated by tree diversity (a measurement of the richness and diversity of trees). However, this impact was not found in adult females. Moreover, the hypothesis that green space structure influences the prevalence of asthma by reducing air pollution was not confirmed in this study, which can be attributed to a variety of causes.

## 1. Introduction

Asthma is a major chronic lung disease characterized by airway inflammation and usually leads to bronchoconstriction and increases mucus production in the airway [1]. The highest prevalence is in children, and most deaths occur in older adults [2]. It has been reported that more than 339 million people have developed asthma, with approximately 417,918 people dying from asthma and approximately 24.8 million disability-adjusted life years (DALYs) attributed to asthma globally in 2016 [2]. Continuous airway inflammation from childhood asthma has a profound influence on adulthood respiratory health and lung function [3], associated, for example, with a higher risk of chronic obstructive pulmonary disease in adulthood [4]. Additionally, asthma can exert multiple adverse impacts on patients, such as increasing anxiety, decreasing self-confidence, and reducing outdoor/physical activities [5]. Therefore, the potential factors that can directly/indirectly lead to asthma have been widely examined, such as genes, ethnicity, family history, maternal lifestyle and diet, age, gender, and income [1,6].

Notwithstanding the complex and genetic characteristics of asthmatics [7], the development of gene–environment interaction theory from an epidemiologic perspective makes alleviating asthma, as well as asthma-like diseases, via the surrounding environment possible [8]. Air pollution has been found to have a negative influence on the asthma-related outcomes of children and adults [9]. For example, higher exposure to nitrogen dioxide (NO_2_) early in life produces a long-term influence on asthma development across approximately 20 years [10]. Similarly, long-term exposure to particulate matter (PM) early in life is likely to increase the risk of subsequent asthma throughout childhood by up to 12 years [11]. The underlying mechanism between air pollution and asthma may be partly explained by oxidative stress [9]. The inhaled polluted air in the airways can help to generate reactive oxygen species and thus can lead to airway hyperreactivity, epithelial cell inflammation, and lung impairment. In addition to the indirect method of inflammation induction, pollution deposition can also directly influence the airway epithelia by producing free reactive oxygen species, which diffuse from the airway surface [12]. Moreover, oxidative stress has been found to correlate with the lower corticosteroid responsiveness of asthmatics [13]. Based on this, exposure to air pollution increases the risk of asthmatics’ failure to deal with inhaled allergens.

There is abundant evidence that has proven that green space plays a significant role in reducing air pollutants, such as PM, NO_2_, and ozone (O_3_) [14,15]. On the one hand, vegetation can absorb gaseous pollutants via its leaf stomata or branch lenticel, and then the absorbed gases are transformed into non-toxic substances through redox reaction to be excreted by roots or stored in inner organs [16]. On the other hand, vegetation can intercept airborne particles and retain them on its surface, and the retained particles are taken away by rain or fallen leaves and twigs [15,17]. Hence, reducing air pollution is usually regarded as an important pathway by which green spaces relieve asthma, as well as asthma-related symptoms [18,19]. Notably, the characteristics of green spaces, especially type, structure, and quantity, create significant differences in their mitigation effects [15,20]. For example, air pollution is negatively correlated with the percentage of tree cover areas and positively with mixing layer heights [15]. Moreover, according to the biodiversity hypothesis, interacting with the natural environment has the potential to improve immunoregulatory circuits and to decrease inflammatory disorders [21]. The microbiota in such a biodiverse environment can promote the diversity of human commensal microbiota, such as the plentifulness of Gammaproteobacteria on the skin, which in turn influences human immunologic tolerance [22]. In other words, besides the mediation effect of air pollution, green spaces can also directly make a difference in the outcomes of asthmatics due to their biodiverse components [19].

Given the research status talked above, the characteristics of green spaces, such as greenness, accessibility, and green types, are commonly used to measure the green space exposure of the asthmatics [23,24,25]. Additionally, the most commonly applied indicators to measure greenness are the Normalized Difference Vegetation Index (NDVI) and tree cover/density [19,26]. It is intriguing that the conclusions on the impact of green spaces on asthma are not consistent or even contrary [27]. For example, some studies have found that greenness negatively correlates with the prevalence of asthma and its related symptoms, whereas others have failed to find a significant correlation between them [28,29,30]. Some have even generated adverse conclusions by using other indicators to describe green spaces [31,32]. This can be partially explained by green spaces having a dual and opposing influence on asthma. Not only can they generate benefits by improving air quality and influencing microbial agents, but they can also cause detrimental effects by producing allergenic pollen and reacting with air pollutants [32,33]. Rather than exploring the multi-pathways between green spaces and asthma (concurrently focusing on the direct and indirect mechanisms between them), most relevant studies have explored only one pathway or partial pathway, such as the association between green spaces and asthma mediated by air quality or green spaces together with air quality [9,19,34]. Considering the possible counteracting effects of different pathways between green spaces and asthma, it is necessary to conduct a more comprehensive analysis to understand the simultaneous multi-pathways between them.

Furthermore, greenness, measured by tree density, has been proven to negatively correlate with asthma in some studies [35], but it does not necessarily mean that such a relationship still exists when other types and proportions of vegetation are taken into account. This is because different green space structures can play different roles in influencing air quality and microbiota. For instance, trees have a better reduction effect on air pollution than shrubs and grass [36], and, compared to the structure of shrubs plus grass, trees together with shrubs or grass have a relatively higher average PM reduction rate (including PM_1_, PM_2.5_, and PM_10_) [37]. In other words, green space structures consisting of different vegetation types (trees–shrubs–grass, trees–grass, trees only, shrubs–grass, etc.) may have different abilities to influence air pollution. Even if the components are the same, such as trees plus shrubs, the effect of green spaces on air pollution in terms of both extent and direction might vary when the ratio of the tree and shrub areas is different. It has been proven that when the tree area is almost equal to the shrub area, the space is more enclosed, and if the shrub area is greater than the tree area, then the space is relatively open [38]. This can subsequently influence the spread of air pollution.

Moreover, the structure of vegetation components can influence microbial diversity [39,40]. First, shrubs and grass can directly impact the soil microclimate, nutrient availability, and pH value through the litter and root exudates [41,42]. Additionally, trees can influence the microorganisms through both the litter and secretion to enter the soil, and the nutrient cascade. In detail, trees can not only reduce the growth, productivity, and diversity of understory vegetation through allelopathy and competition for light, water, and nutrition to indirectly impact the understory vegetation entering the soil [43]; they also directly influence other soil organisms that can have an influence on the soil microorganisms by the nutrient cascade effect [44]. Furthermore, shrubs, grass, and trees can jointly make a difference in microbial diversity by the soil microenvironment, as well as the litter and secretion entering the soil [45]. Thus, the structure of vegetation components can change the prevalence of asthma as a result of changing the microbial condition on a human’s skin and nasal cavity [46]. In sum, green space structures can influence air pollution and microbial diversity, but with different results due to the variation in green types and the proportions of vegetation components, which, as a result, leads to the differences in its influence on the prevalence of asthma. Hence, a better knowledge of the relationship between green space structures and asthma, as well as the potential pathways between them, can help urban planners and policy-makers to reduce the risk and severity of asthma by devising planning schemes and intervention mechanisms.

Considering the reasoning above, this research took Toronto as a case study to explore three questions: (1) Does green space structure have a direct relationship with the prevalence of asthma? (2) Does green space structure influence the prevalence of asthma by reducing air pollution? (3) Is the influence of green space structure on the prevalence of asthma adjusted by related moderators? To this end, the rest of this paper is organized as follows: Section 2 introduces the research materials and analysis methods; the analysis results are presented in Section 3; in Section 4, the underlying causes for the analysis results are discussed, and the paper is ended with conclusions in Section 5.

## 2. Materials and Methods

### 2.1. Research Area

**Figure 1:**
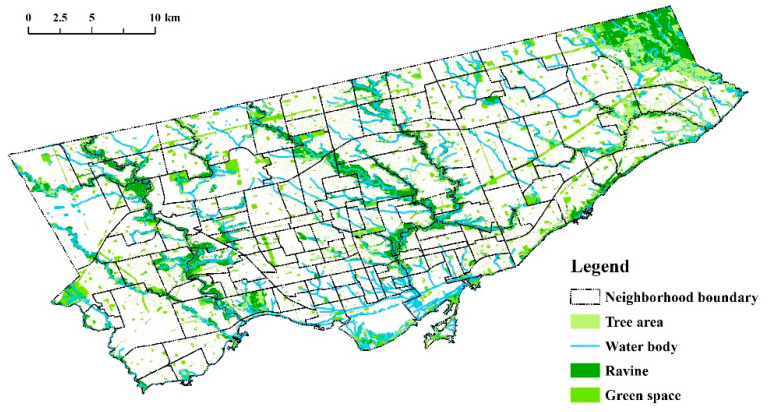
Study area (source: Toronto Open Data).

The research case, i.e., Toronto, is the capital of Ontario province, located on the northwest shore of Lake Ontario in Canada. Plenty of vegetation, various types of green spaces, and the abundant vegetation species in Toronto [47] make the consideration of diverse vegetation structures possible for this research. It covers approximately 630 km^2^ of land, with over 8000 hectares designated as parks (concerning the types of woodlots, destination parks, parkettes, etc.) (Figure 1), and comprises nearly 10 million trees (including approximately 600,000 street trees and 3.5 million park trees) [48]. Moreover, Toronto has a large number of ethnic minorities, and more than half of the population (approximately 51.5%) are the visible minorities, which is higher than the average proportion in Ontario (29.3%) [49]. This means that the generated findings (with statistical significance) in this study will likely suit a wide spectrum of ethnicities and will potentially be a reference for concerned ethnicities in other places or countries. Asthma, as the third most common chronic disease in Canada, has received wide research and attention [50]. Compared to Ontario (15.4 per 100 people in 2016), which has the highest prevalence of asthma in Canada [51], Toronto has a slightly lower prevalence of asthma (14.8 per 100 people in 2016) [52]. In total, there are more than 700 facilities reported to release 25 priority chemicals; volatile organic compounds (VOCs), nitrogen oxide (NOx), and PM2.5, which are the top three air pollutants, accounted for 75.13%, 19.96%, and 3.9% of the total priority substances released into the air in 2016, respectively [53].

### 2.2. Research Design

**Figure 2:**
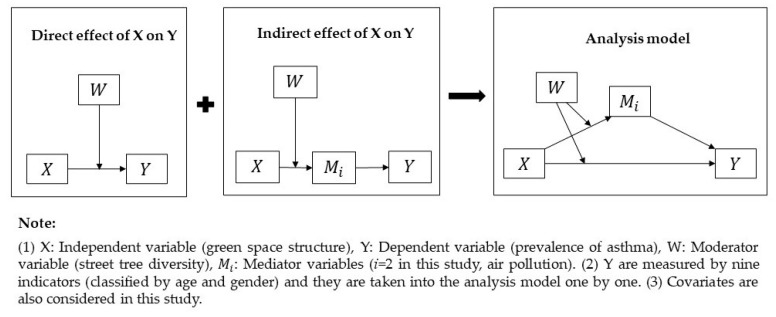
Research framework.

In light of the research questions, two potential influencing pathways were explored in this study (Figure 2). In detail, the direct pathway refers to the influence of green space structure per se on the prevalence of asthma, while the indirect pathway points to the mediators’ impact on the relationship between green space structure and the prevalence of asthma. In particular, the former is based on the biodiversity hypothesis, and it supposes that green space structure plays a role in the prevalence of asthma via its micro-components (diverse microbiota). The latter is formulated by the hypothesis that green space structure affects the prevalence of asthma mediated by a reduction in air pollution. Moreover, the related moderator was also taken into account in the analysis model with controlling covariates. In general, this study was conducted at the population level, and the neighborhood was treated as the research unit. The values of all the variables were standardized before analysis using SPSS 20 v. PROCESS 3.4 (Andrew F. Hayes, Ohio State University, USA) (downloaded from http://processmacro.org/download.html (accessed on 10 August 2019)).

### 2.3. Variables Measurement

#### 2.3.1. Dependent Variables

Previous studies have reported that the triggers of child and teenage asthma and adult asthma are probably different, and the gender proportions of asthmatics at different ages can also cause differences [1]. In other words, even if the surrounding environment is the same, it still can generate different influences on the prevalence of asthma among different age and gender groups. Considering this, we classified asthmatics (including all ages and genders) into different groups by their age and gender (Table 1 and Appendix A Table A1) and analyzed them separately (Figure 2). This contributed to a better understanding of how the selected impact factors play different roles among the various asthmatic groups.

#### 2.3.2. Independent Variable

The green space structure is measured by the ratio of vegetation components in this study. The data of the vegetation types (trees, shrubs, and grass) were derived from multispectral satellite imagery with the assistance of light detection and ranging (LiDAR) information and classified automatically by following certain classification model logic (for the detailed analysis methodology, please refer to the 2018 Land Cover Documentation released by Toronto Parks, Forestry and Recreation [54]). We combined the shrub class with the grass class to form the shrub–grass class to reduce the classification bias. In other words, the vegetation components of trees and shrub–grass were distinguished in this study, and based on this, the indicator of the ratio of trees to shrubs–grass was calculated to measure green space structure (Table 1). Notably, the original data of the trees, shrubs, and grass were not presented by the unit of the neighborhood. Using ArcGIS 10.3, they were extracted from the Toronto land cover dataset first according to the class codes, and the tool of Intersect was applied to connect their geographic locations with neighborhood shapes (transformation of projection coordinates was performed during this process). Then, the areas of trees, shrubs, and grass at the neighborhood level were, respectively, summarized in Excel 2007. After this, the ratio of trees to shrubs–grass in each research unit could be calculated (Appendix A Table A1).

#### 2.3.3. Mediator Variables

Inhaled substances and particles that may provoke allergic reactions or irritate the airways are regarded as the strongest risk factors for developing asthma [2], and many of them, such as NO_2_, SO_2_, VOCs, and O_3_, as well as PM, also have adverse impacts on potential asthmatics [9]. Hence, considering the ecological benefits of green space structure in improving air quality, we took the comprehensive indicator of total pollutants released into the air (including 25 priority chemicals) as one of the possible mediators between green space structure and asthma (Table 1 and Appendix A Table A1). Additionally, ultrafine particles (UFPs) have been reported to cause serious detriments to public respiratory health for a relatively long time through directly permeating cell membranes and inducing severe epithelial damage and alveolar macrophage chemotaxis [55,56]. Therefore, the indicator of UFPs was considered as another possible mediator (Table 1). The data of UFPs were derived from Sabaliauskas et al. [57], and we obtained the authors’ permission. It is important to note that although UFPs were measured in 2008 and were inconsistent with the year of asthma and green spaces measured (Appendix A Table A1), such inconsistency can still be accepted. As the average change rate of total pollutants released into the air in Toronto from 2012 to 2018 was 2% (negative value, calculation based on the Toronto Annual ChemTRAC Reports [58]), this means that air quality did not change significantly during this period. UPFs, as a component of air pollutants, likely experience a similar change trend.

#### 2.3.4. Moderator Variable

Tree species play a crucial role in improving air quality due to their morphological characteristics [15]. For example, compared to bushy coniferous species, broad-leaf trees have a higher ability to capture PM [17]. Similarly, compared to leathery- and smooth-leaf trees, those with hairy and rough leaves are more successful in reducing particle concentrations [17]. In other words, tree species can influence the indirect pathway between green space structures and asthma through altering the reduction effect of green space structure on air pollution. Additionally, vegetation diversity/richness has been found to potentially enhance the reduction effect on PM by improving the greenness quantity of the leaf area [59]. For the same structure of vegetation components, the richer the vegetation species are, the better their ability to reduce PM [37]. Moreover, various vegetation species have the potential to generate different levels of pollen allergenicity [60] and microbial communities [61,62], which probably influences the direct mechanism of green space structures on the prevalence of asthma via microbial diversity. Considering that this study analyzed by the unit of the neighborhood, it was impossible to determine all of the vegetation species (including trees, shrubs, and grass/herbs) in each neighborhood to calculate the vegetation diversity/richness. Hence, the indicator of (street) tree diversity was applied to partially reflect the diversity/richness of the vegetation in the study area (Table 1). Similar to the trees, shrubs, and grass, the original data of tree species needed to be attached to the neighborhood shapes first. Thus, the process methods were the same as those mentioned above, but transformation of the projection coordinates was not necessary during this process. It is worth noting that while tree species are plentiful, they vary by neighborhood, and some of them exist in several neighborhoods. Therefore, categorizing tree species on a more general level (genus) is necessary before matching them to neighborhood units, which can facilitate the calculation of tree diversity. For the detailed calculation method, please refer to Appendix A Table A1.

#### 2.3.5. Covariates

Asthma is a complex chronic disease, which can be triggered by multiple factors, including asthma-related genes, indoor and outdoor allergens, tobacco smoke, chemical irritants, and pollen [2]. Since this research was conducted at the population level, individual-level factors were not taken into consideration. Regarding existing findings and data accessibility, factors such as greenness (measured by the percentage of green space), income (measured by average total income), number of family members (measured by average household size), and ethnic group (measured by the percentage of total visible minorities) that possibly influence the prevalence of asthma were selected as the covariates [1,6,63] (Table 1). Therein, the indicators of sibling numbers, family size, etc., were applied to measure the influence of sibship size on asthma or wheezing in previous research, since they are considered as a potential protective factor for allergy-related issues (some research defined this phenomenon as the “sibling effect”) [64]. Different from children-targeted studies, our research additionally included adults. Compared to the number of siblings, family size might be a more appropriate indicator. Therefore, we took the obtained data of household size as a confounder that potentially influences the prevalence of asthma to be controlled. Notably, all of the covariates were calculated at the neighborhood level (Appendix A Table A1).

## 3. Results

**Figure A1:**
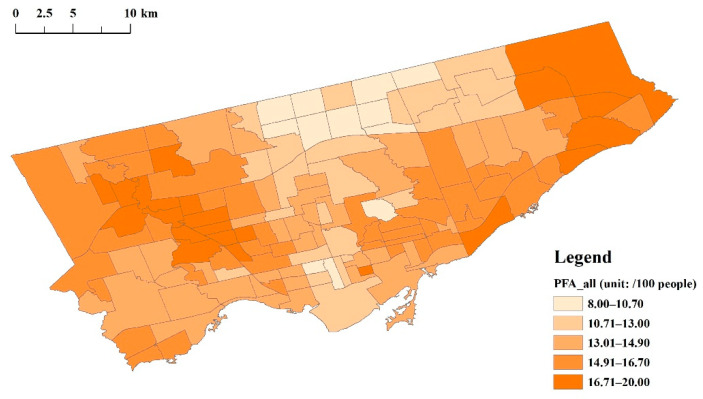
Distribution of the prevalence of female asthmatics at all ages (PFA_all).

**Figure A3:**
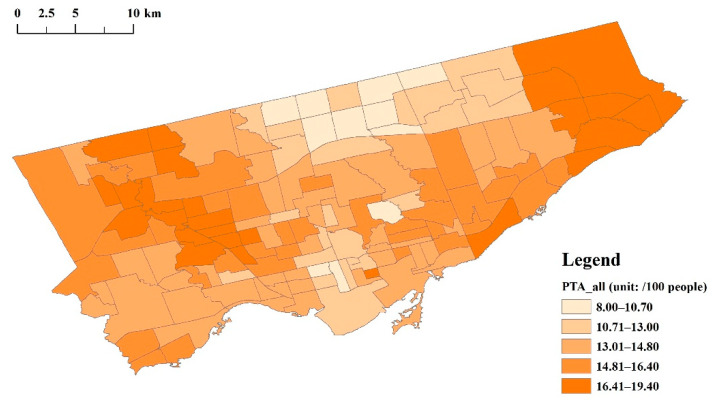
Distribution of the prevalence of total asthmatics at all ages (PTA_all).

**Figure A4:**
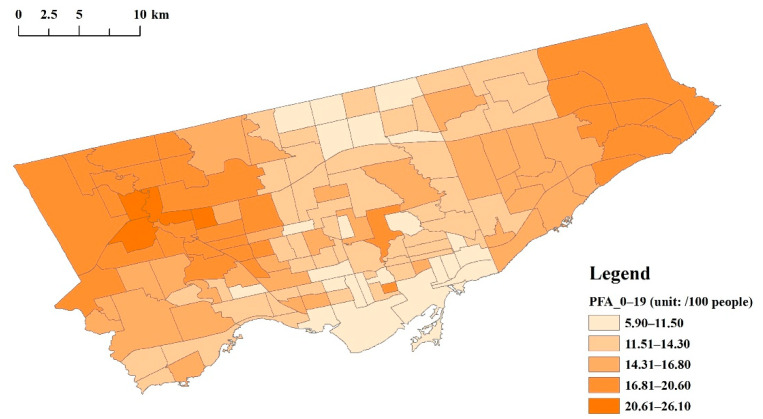
Distribution of the prevalence of female asthmatics aged 0–19 years (PFA_0–19).

**Figure A5:**
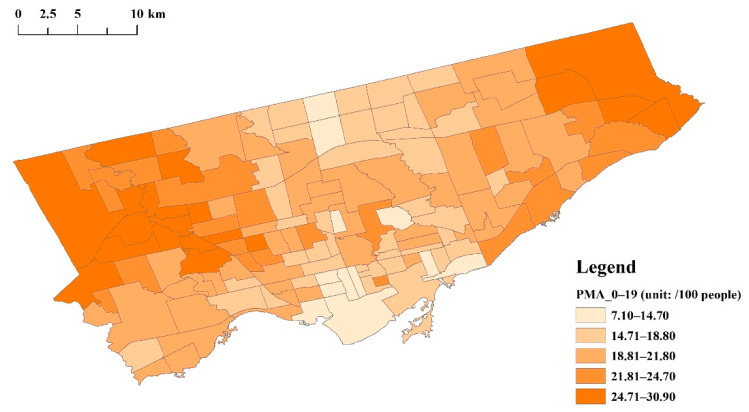
Distribution of the prevalence of male asthmatics aged 0–19 years (PMA_0–19).

**Figure A6:**
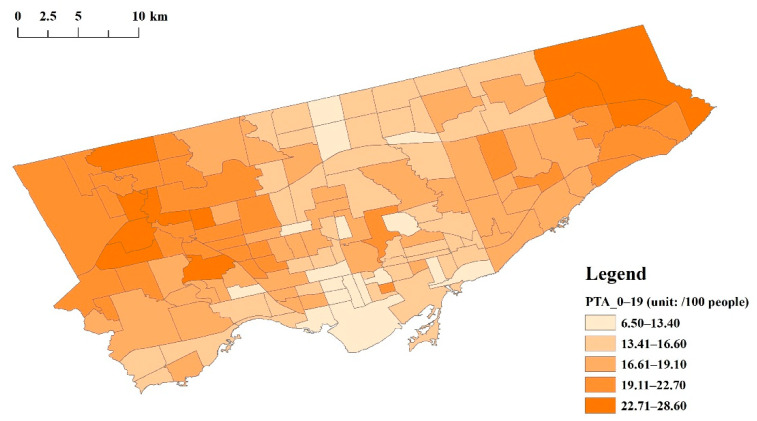
Distribution of the prevalence of total asthmatics aged 0–19 years (PTA_0–19).

**Figure A7:**
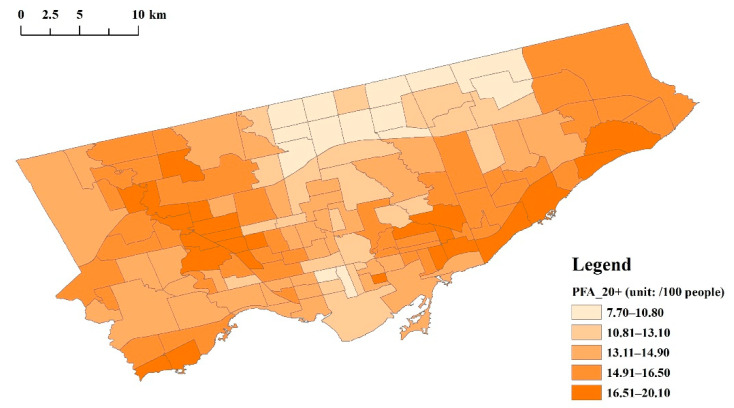
Distribution of the prevalence of female asthmatics aged 20 years and above (PFA_20+).

**Figure A8:**
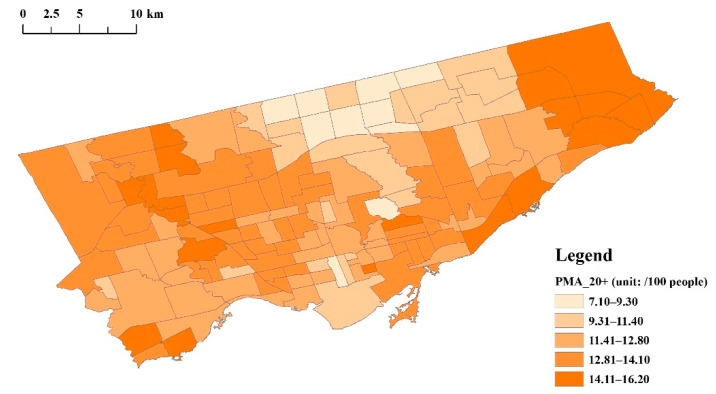
Distribution of the prevalence of male asthmatics aged 20 years and above (PMA_20+).

**Figure A9:**
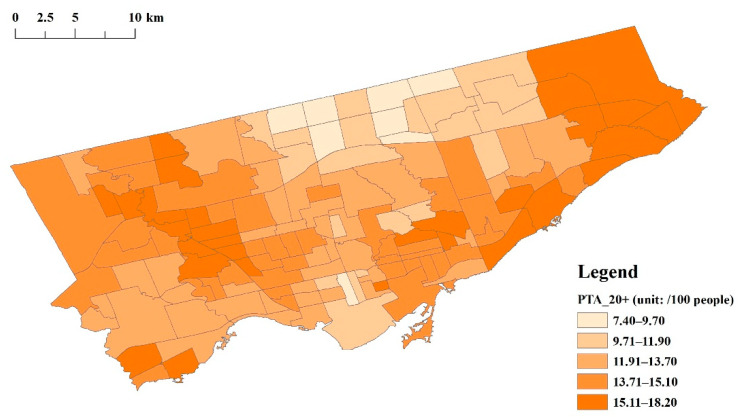
Distribution of the prevalence of total asthmatics aged 20 years and above (PTA_20+).

**Figure A10:**
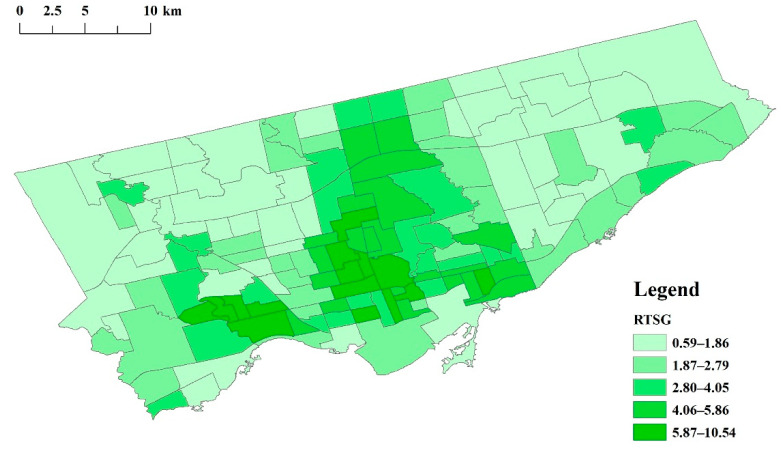
Distribution of the ratio of trees to shrubs–grass (RTSG).

**Figure A11:**
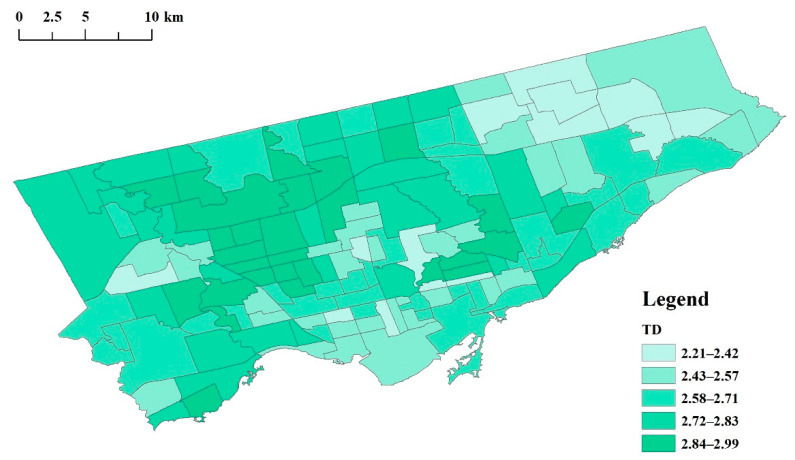
Distribution of tree diversity (TD).

**Figure A12:**
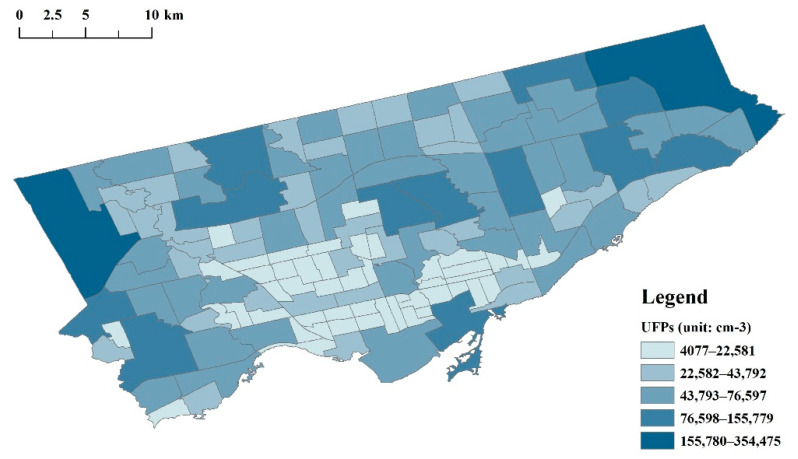
Distribution of ultrafine particles (UFPs).

**Figure A13:**
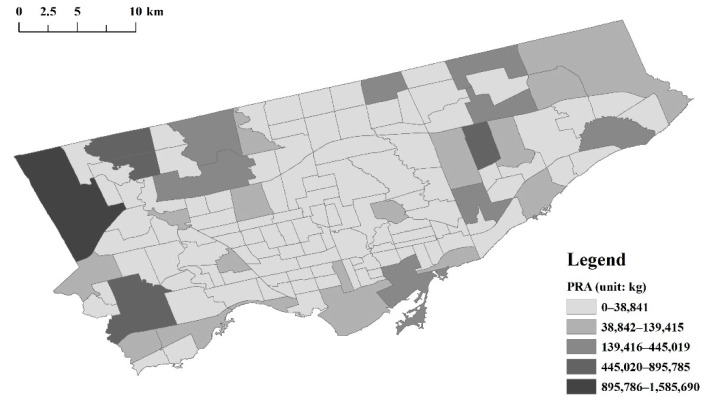
Distribution of pollutants released into the air (PRA).

As described in Table 2, the prevalence of asthma varied across age and gender. Compared to adults, children had a higher risk of developing asthma. For people aged 0–19 years, male asthmatics accounted for the majority, while for those aged 20 years and above, there were more female than male asthmatics. Such characteristics of the prevalence of asthma are consistent with existing findings [1]. The mean ratio of trees to shrubs–grass indicates that most neighborhoods were able to provide canopy space for outdoor activities [65]. From the maximum and minimum values of UFPs and pollutants released into the air (PRA), we can infer that significant differences in air pollution exist among the neighborhoods. However, tree diversity did not vary significantly. The relatively high value of percentage of green space (POGS) indicates that the whole population had potential for high green space exposure. Moreover, the rather high mean percentage of total visible minorities (PTVM) reflects that most neighborhoods had highly diverse ethnic groups. To intuitively present the independent, dependent, mediator, and moderator variables, their spatial distributions at the neighborhood level are depicted in Appendix A Figure A1, Figure A3, Figure A3, Figure A4, Figure A5, Figure A6, Figure A7, Figure A8, Figure A9, Figure A10, Figure A11, Figure A12 and Figure A13.

As the partial influencing pathway (Figure 2), the correlations between green space structure and air pollution, as well as the probable moderator (tree diversity), were analyzed first. As shown in Table 3, the ratio of trees to shrubs–grass (RTSG) was negatively related to UFPs and had no significant association with the PRA. On the contrary, the relationship between the POGS and the PRA was negative, but the POGS had no significant association with UFPs. Moreover, tree diversity (TD) did not play a role in influencing UFPs or the PRA (neither coefficient of TD was statistically significant), and could not adjust the relationship between the RTSG and UFPs or the PRA (neither coefficient of the interaction between the RTSG and TD (Int_1) was statistically significant). Intriguingly, total income (TI) had a positive impact on UFPs but did not influence the PRA.

The overall analysis results are outlined in Table 4. In general, the RTSG had a significantly negative relationship with asthmatics aged 0–19 years, no matter if they were female or male, while it had no impact on adult asthmatics. Unexpectedly, neither UFPs nor the PRA were found to correlate with the prevalence of asthma. Interestingly, although TD did not directly influence the prevalence of asthma, it could make a difference by adjusting the relationship between the RTSG and asthma. In other words, moderated by TD, the influence of the RTSG on asthma could be statistically significant. However, Int_1 was statistically significant only in adult male asthmatics. Except for the POGS, the other three selected covariates played a role in the prevalence of asthma. In particular, household size (HS) and the PTVM influenced all asthmatic groups with statistical significance, while TI was only significant in the adult female asthmatics.

UFPs and the PRA did not correlate with the prevalence of asthma in this study, which indicates the RTSG did not affect asthma mediated by UFPs or the PRA. However, the RTSG had the potential to directly influence asthma (Table 4). Detailed conditional effects analysis contributed to a better understanding of how the RTSG, as well as TD, influences the prevalence of asthma at different threshold intervals. As discussed above, the RSTG did not statistically correlate with adult asthmatics, but when moderated by TD (value in the 84th percentile), it had a significant protective influence on asthmatics (Table 5). However, the moderation effect of TD on the RSTG only functioned significantly for adult male asthmatics. Moreover, despite the Int_1 coefficient not being statistically significant in asthmatics aged 0–19 years, this does not mean that TD cannot adjust its association with the RSTG. As depicted in Table 5, compared to the conditional effect of the RSTG on asthmatics aged 0–19 years, with TD being in the 50th percentile, it was significantly higher when TD was in the 84th percentile. In other words, from the whole perspective, TD did not moderate the relationship between the RSTG and the prevalence of asthmatics aged 0–19 years, but there was a significant moderation effect when TD reached a certain threshold interval (84th percentile in this study). Collectively, the RSTG can play a direct role in developing asthma for those aged 0–19 years. Additionally, it could also influence the prevalence of adult male asthma when moderated by TD. However, the moderation effect of TD on the RSTG only existed in the 84th percentile in this study. It is noteworthy that the RSTG did not influence adult female asthma; even when moderated by relatively high TD, the influence of the RSTG was still not significant.

## 4. Discussion

In general, the hypothesis of the direct pathway, as illustrated in Figure 2, was confirmed in this study, but only in asthmatics aged 0–19 years. For adult male asthmatics, only when the RSTG was moderated by the 84th percentile TD were they influenced by the RSTG. In other words, the ratio of trees to shrubs–grass has the potential to directly reduce the prevalence of asthma for residents aged 0–19 years, whether it is moderated by tree diversity or not. However, it did not exert a reduction effect on the prevalence of asthma for people aged 20 years and above until moderated by the relatively high tree diversity. However, this protective influence did not exist in adult female asthmatics. This might be due to the difference in green space use for different groups. As discussed above, the direct pathway between the ratio of trees to shrubs–grass and asthma is based on the hypothesis of biodiversity, which implies that actual green space use is the precondition for green spaces to influence asthma prevalence via diverse microbiota. Specifically, only when people are physically exposed to green spaces is it possible for the micro-composition of green spaces to improve the microbiota on human skin [46]. For children, adolescents, and adults, the esthetic attributes of green spaces, such as the richness and diversity of the vegetation, are important factors that influence the duration and frequency of their green space use [66]. Nevertheless, women are less likely to use green spaces if they are perceived as being unsafe [67], while men are not easily influenced by unsafe perceptions [68]. Additionally, the frequency of males’ usage of green spaces increases with their age, up until 80 years old, whereas this phenomenon does not exist in women [69]. Given this, whether or not the ratio of trees to shrubs–grass directly exerts an influence on asthma seems to partially depend on whether people use green spaces or not.

Besides, asthma is a complex chronic respiratory disease. Besides the factors that we focused on at the population level in this study, there are many other individual-level factors that impact the prevalence of asthma, such as genes and lifestyle [1]. In contrast to atopy and family asthma history as the major triggers for childhood asthma, adult asthma mainly correlates with the female gender, smoking habits, and low socioeconomic status [70]. In other words, even if the ratio of trees to shrubs–grass can play a protective role in the process of developing asthma, these individual adverse factors might offset such effects. Moreover, the protective roles of biodiversity, which are performed by increasing human microbiota, may be more effective for children and adolescent asthmatics, since they are susceptible to allergic atopy, and diverse microbials obtained from green spaces have the potential to improve their immunoregulatory capacity, which possibly protects them from developing chronic inflammatory diseases [71]. As a result, the ratio of trees to shrubs–grass has a decreasing effect on the prevalence of asthma in those aged 0–19 years and such an influence can be enhanced by tree diversity. However, it makes no difference in the prevalence of adult asthma on the whole and needs extra adjustment/enhancement of the moderator (tree diversity) to make the influence on adult male asthma significant.

Unexpectedly, we did not find a statistically significant relationship between UFPs or the PRA and the prevalence of asthma. In other words, the hypothesis of the indirect influencing pathway was not confirmed in this study. Several possibilities can explain such results. First of all, unlike previous studies analyzing certain single pollutants’ influences on asthma, this study used the PRA, a comprehensive indicator. This indicator refers not only to the pollutants that have been reported to positively correlate with asthma (NO_2_, PM2.5, etc.), but also contains other substances that possibly have no significant relationship with asthma (chloroform, dichloromethane, etc.). In other words, neighborhoods with high total amounts of priority substances released into the air do not necessarily have high concentrations of NO_2_, PM2.5, etc., which may be the cause of the nonsignificant relationship between the PRA and asthma. Second, the value of UFPs used in this study was probably lower than the value that it should be in reality. Existing studies suggest that the UFPs concentration has a negative correlation with ambient temperature and it is relatively higher in winter months [72,73]. However, the UFPs data applied in this study were measured in the summer, when the temperature was 25–32 °C [57]. Therefore, compared to the actual UFPs exposure, the measured UFPs concentration had probably not yet reached the threshold to influence asthma. Finally, the time inconsistency between the air pollution data, especially the UFPs and asthma data, may also be important. Although the annual change rate of total pollutants released into the air was small, it was still different from that in the year when the data of asthma were collected. Thus, the time bias of these variables might partially explain the uncorrelated relationship between air pollution and the prevalence of asthma.

The analysis also found that PTVM has a negative relationship with asthma prevalence, which resonates with the existing conclusions [74,75]. On the one hand, previous studies have shown that the duration of residence of immigrants in developed countries is positively associated with asthma prevalence, as well as asthma-related outcomes [76,77], and compared with the native-born people, immigrants, as well as their children, have lower asthma morbidity [78]. This might be due to early life exposure in their home countries that can continuously protect them [74]. On the other hand, it has also been found that the prevalence of asthma differs between ethnic groups [79,80]. For example, Hispanic individuals have a lower risk of developing asthma than non-Hispanic White and Black individuals [81]. Chaldean and Arab individuals have a lower prevalence of asthma than non-Middle Eastern white and African American individuals [82]. A great deal of evidence has suggested that genetic variation and gene–environment interactions are moderately accountable for the ethnic discrepancy in asthma morbidity and prevalence [83,84]. As discussed above, the negative association between PTVM and asthma prevalence possibly results from the relatively short residence time and low asthma-related genes of visible minorities in Toronto compared with non-minorities. Household size was found to be positively related to the prevalence of asthma, which aligns with the conclusions from Weitzman et al. [85] using the same indicators, but contrasts the findings obtained by using the indicator of sibling numbers [86]. This discrepancy might be due to the different measurements of sibship size and targeted groups.

Household income was negatively related to the prevalence of adult asthma, especially female asthma, but had no evident influence on asthmatics aged 0–19 years. These findings can be partially explained by three aspects. First, as discussed above, the RTSG or the RTSG combined with TD can negatively influence the prevalence of total asthmatics aged 0–19 years and male asthmatics aged 20+ years, whereas this suppression effect of green space did not affect female asthmatics aged 20+ years. In other words, regarding the low-income group, the adverse influence of low income on total asthmatics aged 0–19 years and male asthmatics aged 20+ years can be alleviated or offset by the benefits of green spaces, but not for female asthmatics aged 20+ years. As a comparison, income seems to be more significant for female asthmatics aged 20+ years, and with the increase in household income, the impacts of the lower economic status are mitigated. Second, a series of studies have found that, compared to male asthmatics, female asthmatics tend to spend more on medication and become hospitalized, even with a similar asthma severity as male asthmatics [87,88] as they have a more sensitive perception of asthma and are more likely to report more severe asthma-related symptoms [89]. As a result, the preference of taking healthcare use-related actions probably results in income level becoming more significant for female asthmatics than male asthmatics. Correspondingly, high income can exert a negative influence on the prevalence of asthma in females. It is equally important that asthma prevalence is also possibly influenced by smoking and stress [1,70,90]. As has been revealed, chronic stress is positively correlated with interleukin production and eosinophil counts, which implicates the inflammatory process in asthma, while higher family savings contribute to reducing interleukin production and eosinophil counts [91]. Moreover, stress can promote tobacco use [92], and, compared with males, females are more likely to smoke when managing stress [93]. However, household income is negatively related to nicotine dependence and chronic stress, and it can moderate the positive relationship between stress and smoking [92]. Therefore, household income possibly influences adult asthmatics through tobacco use and chronic stress, and higher income can provide alleviation for adult asthmatics (especially for female asthmatics).

Greenness, measured by the percentage of green space, is not associated with asthma, as revealed, which is in line with some existing studies. For example, Dadvand et al. found that greenness, measured by the Normalized Difference Vegetation Index (NDVI), is not associated with the prevalence of asthma, while living close to parks causes a significant difference in the prevalence of current asthma [30]. Nevertheless, there are other different conclusions. Zeng et al. found that NDVI negatively correlates with the prevalence of asthma and wheezing [28]. Contrarily, Andrusaityte et al. reported that greenness is positively related to the risk of asthma [24]. The inconsistencies among different studies on the relationship between greenness and asthma may be due to the differences in measurements, locations, and scales of greenness. For instance, Dadvand et al. (Spain) measured greenness within the buffer area of respondents’ geocoded addresses, while Zeng et al. focused on the greenness surrounding schools, and Andrusaityte et al. (Lithuania) used the mean NDVI within the buffer areas of participants’ homes. In this study, however, instead of concentrating on the surrounding greenness of certain land use types, greenness was calculated at the neighborhood level and measured by the percentage of green space (total vegetation).

It is important to note the potential limitations in this study. First, the vegetation types of trees, shrubs, and grass were classified automatically with the assistance of LiDAR information, which might have resulted in slight differences from the vegetation structure in reality. Although shrubs and grass were treated as one category in this study, classification bias probably still influenced the analysis results. Moreover, the time inconsistencies (data were generated in different years) among the data of air pollution, green spaces, and asthma may have also cause differences in the analysis results. For instance, the reason that a mediation effect of air pollution on the relationship between the ratio of trees to shrubs–grass and the prevalence of asthma was not found in this study might be partially attributed to this. Additionally, the analysis presented in this study was based at the population level, so many asthma-related impact factors at the individual level were not considered. The co-effects of these positive and adverse factors can jointly exert their influence upon the development of asthma. Thus, without accounting for individual-based impact factors, the conclusions generated in this study may have overemphasized or overlooked the effects of the selected factors upon asthma prevalence. Finally, some studies have suggested that the geographic context is also important. Studies conducted in different locations might generate different or even contrary conclusions on the same issues [25]. Therefore, the limitations of this research, as explained above, need to be kept in mind when applying the conclusions to other research contexts or practices.

## 5. Conclusions

This study mainly explored the two pathways between green space structure (measured by the ratio of trees to shrubs–grass) and the prevalence of asthma (categorized into nine detailed indicators by gender and age) based on controlling the related covariates at the population level. In particular, the direct pathway is based on the biodiversity hypothesis, and the indirect pathway is related to the green structure influencing the prevalence of asthma by reducing air pollution. According to the analysis, the direct pathway was statistically significant for those aged 0–19 years, while the indirect pathway was not well justified. Furthermore, green space structure can play a protective role for male asthmatics aged 20 years and above when moderated by a higher tree diversity, whereas this effect did not exist in female asthmatics aged 20 years and above, even when moderated by higher tree diversity. Additionally, green space structure can generate a protective effect for asthmatics aged 0–19 years, whether female or male, and such a protective influence can be enhanced by a higher tree diversity.

## Figures and Tables

**Table 1:** The description of variables.

Variables			Description	
Category	Indicators	Abbreviations		
Dependent variables	Prevalence of total asthmatics at all ages	PTA_all	Numbers of asthmatics per 100 people (including male, female, and both sexes) at all ages, 0–19 years, and 20+ years	Prevalence of asthma
	Prevalence of male asthmatics at all ages	PMA_all		
	Prevalence of female asthmatics at all ages	PFA_all		
	Prevalence of total asthmatics aged 0–19 years	PTA_0–19		
	Prevalence of male asthmatics aged 0–19 years	PMA_0–19		
	Prevalence of female asthmatics aged 0–19 years	PFA_0–19		
	Prevalence of total asthmatics aged 20 years and above	PTA_20+		
	Prevalence of male asthmatics aged 20 years and above	PMA_20+		
	Prevalence of female asthmatics aged 20 years and above	PFA_20+		
Independent variable	Ratio of trees to shrubs–grass	RTSG	Ratio of tree areas to shrub and grass areas, an indicator of the vertical component characteristic of vegetation	Green space structure
Mediator variables	Ultrafine particles	UFPs	Ultrafine particles with diameters mainly between 8 and 300 nm, the majority of measuring sites concern railroads, expressways, arterial road, etc.	Air pollution
	Pollutants released into the air	PRA	Total pollutants released into the air, total amount of priority substances released into the air, a comprehensive indicator, including different pollutant chemicals, such as volatile organic compounds (VOCs), NOx, and PM2.5	
Moderator variable	(street) Tree diversity	TD	Tree diversity, a measurement of the richness and diversity of street trees	Biodiversity
Covariates	Percentage of green space	POGS	Percentage of green space at the neighborhood level, reflection of the quantity of total vegetation	Greenness
	(average) Total income	TI	Total income, the sum of certain incomes of the statistical unit for the population aged 15 years and over in private households (at the neighborhood level)	Economics
	(average) Household size	HS	Household size, the number of persons in a private household, a characteristic of dwelling (at the neighborhood level)	
	Percentage of total visible minorities	PTVMP	Percentage of total visible minorities, indirect reflection of genetic diversity; Employment Equity Act defines visible minorities as “persons, other than Aboriginal peoples, who are non-Caucasian in race or non-white in color”	Demographics

**Table 2:** Descriptive statistics (before standardization).

Variables (Units)		*N*	Minimum	Maximum	Mean	Std. Deviation
Y	PTA_all (/100 people)	140	8.00	19.40	14.38	2.24
	PMA_all (/100 people)	140	8.00	19.40	14.27	2.20
	PFA_all (/100 people)	140	8.00	20.00	14.50	2.35
	PTA_0–19 (/100 people)	140	6.50	28.60	17.53	3.61
	PMA_0–19 (/100 people)	140	7.10	30.90	20.18	4.07
	PFA_0–19 (/100 people)	140	5.90	26.10	14.74	3.27
	PTA_20+ (/100 people)	140	7.40	18.20	13.51	2.00
	PMA_20+ (/100 people)	140	7.10	16.20	12.53	1.73
	PFA_20+ (/100 people)	140	7.70	20.10	14.41	2.34
X	RTSG (N/A)	140	0.59	10.54	3.16	1.97
M	UFPs (cm^−3^)	140	4077	354,475	43,447.25	43,762.34
	PRA (kg)	140	0	1,585,690	58,944.02	184,007.30
W	TD (N/A)	140	2.21	2.99	2.66	0.18
C	POGS (N/A)	140	0.12	0.67	0.37	0.12
	TI ($)	140	25,989	308,010	55,248.49	38,738.60
	HS (persons/household)	140	1.50	3.40	2.49	0.40
	PTVM (N/A)	140	0.12	0.95	0.46	0.22
Valid *N*		140				

Note: (1) Y, X, M, W, and C represent the dependent, independent, mediator, moderator, and covariate variables, respectively. (2) “Valid *N*: 140” means that there were a total of 140 samples (research units) in this study, and all of the samples were considered in the analysis (no one was omitted). (3) “N/A” means indicators are dimensionless.

**Table 3:** Coefficients of variables (outcome variable: Mediators).

Coefficients	Variables						
	X	W	I	C			
	RTSG	TD	Int_1	POGS	TI	HS	PTVM
UFPs	−0.31 **	−0.04	0.10	0.07	0.23 *	0.20	0.17
PRA	−0.10	0.05	−0.01	−0.32 **	0.16	0.22	0.11

Note: (1) I represents the interaction of the moderator (W) and independent (X) variables, and Int_1 refers to the interaction between the RTSG and TD. (2) C includes the variables of POGS, TI, HS, and PTVM. (3) * *p* ≤ 0.05 and ** *p* ≤ 0.01.

**Table 4:** Coefficients of variables (outcome variable: Prevalence of asthma).

Coefficients		Variables								
		X	M		W	I	C			
		RTSG	UFPs	PRA	TD	Int_1	POGS	TI	HS	PTVM
At all ages	Both sexes	−0.19	0.02	0.01	0.07	−0.19 *	0.12	−0.23 *	0.46 ***	−0.53 ***
	Male	−0.19	0.03	0.02	0.08	−0.18 *	0.10	−0.11	0.56 ***	−0.48 ***
	Female	−0.17	0.01	0.01	0.07	−0.18	0.13	−0.33 **	0.35 ***	−0.54 ***
At 0–19 years	Both sexes	−0.27 **	0.01	0.02	0.09	−0.07	0.12	−0.06	0.53 ***	−0.27 **
	Male	−0.25 *	0.00	0.05	0.10	−0.03	0.14	−0.05	0.54 ***	−0.29 **
	Female	−0.28 *	0.02	−0.02	0.06	−0.12	0.09	−0.08	0.49 ***	−0.24 *
At 20+ years	Both sexes	−0.13	0.03	0.00	0.06	−0.20 *	0.10	−0.30 **	0.36 ***	−0.63 ***
	Male	−0.15	0.07	−0.01	0.05	−0.21 *	0.03	−0.15	0.43 ***	−0.63 ***
	Female	−0.11	−0.01	0.02	0.06	−0.19	0.14	−0.39 ***	0.29 **	−0.60 ***

Note: (1) Int_1 means the interaction between the RTSG and TD. (2) C includes the variables of POGS, TI, HS, and PTVM. (3) * *p* ≤ 0.05; ** *p* ≤ 0.01; *** *p* ≤ 0.001. (4) Level of confidence for all confidence intervals in the output: 95.0000. (5) The dependent variables PTA_all, PMA_all, PFA_all, PTA_0–19, PMA_0–19, PFA_0–19, PTA_20+, PMA_20+, and PFA_20+ were taken into the analysis model one by one.

**Table 5:** Conditional direct effect(s) of the RSTG on the prevalence of asthma.

TD Percentiles	Effects								
	At All Ages			At 0–19 Years			At 20+ Years		
	Both sexes	Male	Female	Both Sexes	Male	Female	Both Sexes	Male	Female
16th	0.02	0.01	0.03	−0.19	−0.21	−0.14	0.10	0.09	0.10
50th	−0.19	−0.20	−0.17	**−0.27**	**‒0.25**	**‒0.28**	−0.13	−0.15	−0.11
84th	**−0.39**	**−0.39**	**−0.37**	**−0.35**	**–0.29**	**–0.41**	**−0.35**	**−0.38**	−0.31

Note: (1) Conditional direct effect(s) of the RSTG on the prevalence of asthma: C_RSTG_ + C_Int_1_ * TD, where C_RSTG_ is the coefficient of the RSTG, C_Int_1_ is the coefficient of Int_1, and TD is the tree diversity. (2) Bold values represent *p* ≤ 0.05. (3) Level of confidence for all confidence intervals in the output: 95.0000. (4) TD values in conditional tables are the 16th, 50th, and 84th percentiles. (5) The dependent variables of PTA_all, PMA_all, PFA_all, PTA_0–19, PMA_0–19, PFA_0–19, PTA_20+, PMA_20+, and PFA_20+ were taken into the analysis model one by one.

**Figure A2:**
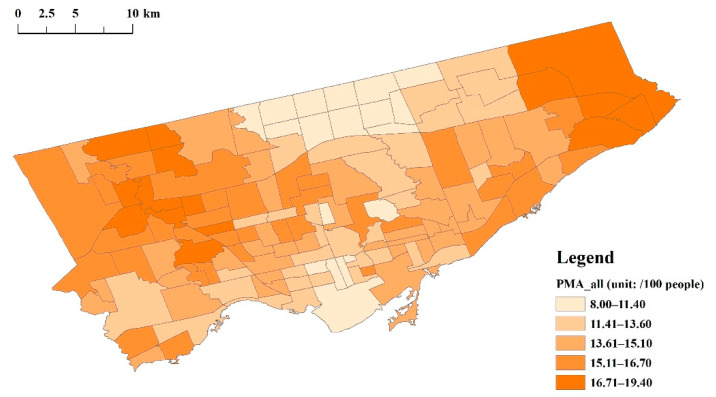
Distribution of the prevalence of male asthmatics at all ages (PMA_all).

## Appendix A

**Table A1:** Variables and requisite data.

Variables		Description		Data			Calculation
Category	Indicators			Requisite Data	Source	Time	
Dependent variables	PTA_all	Numbers of asthmatics per 100 people (including male, female, and both sexes) at all ages, 0–19 years, and 20+ years	Prevalence of asthma	Number (/100) of total, male, and female asthmatics at all ages at the neighborhood level	Ontario Community Health Profiles Partnership	2016–2017	N/A
	PMA_all						
	PFA_all						
	PTA_0–19			Number (/100) of total, male, and female asthmatics aged 0–19 years at the neighborhood level			
	PMA_0–19						
	PFA_0–19						
	PTA_20+			Total asthmatics, female and male asthmatics, total population, and females and males at neighborhood level (aged 20+ years)			Total asthmatics ∗ 100/total population (aged 20+)
	PMA_20+						Male asthmatics ∗ 100/total males (aged 20+)
	PFA_20+						Female asthmatics ∗ 100/total females (aged 20+)
Mediator variables	UFPs	Ultrafine particles with diameters mainly between 8 and 300 nm; the majority of measuring sites concern railroads, expressways, arterial road, etc.	Air pollution	Mean particle number concentrations (cm^–3^) at the neighborhood level	Sabaliauskas et al. (2015)	2008	N/A
	PRA	Total pollutants released into the air, total amount of priority substances released into the air, a comprehensive indicator, including different pollutant chemicals, such as volatile organic compounds (VOCs), NOx, and PM2.5		Pollutants released into the air (kg) at the neighborhood level	Toronto Social Development, Finance and Administration	2012	N/A
Independent variable	RTSG	Ratio of tree areas to shrub and grass areas, an indicator of the vertical component characteristic of vegetation	Green space structure	Tree areas and shrub areas at the neighborhood level	Toronto Parks, Forestry and Recreation	2018	Tree areas/(shrub areas + grass areas)
Moderator variables	TD	Tree diversity, a measurement of the richness and diversity of street trees	Biodiversity	Street tree species at the neighborhood level		2017	
Covariates	POGS	Percentage of green space (total vegetated areas) at the neighborhood level, reflection of the quantity of total vegetation	Greenness	Tree, shrub, grass, and neighborhood areas		2018	(tree areas + shrub areas + grass areas)/neighborhood areas
	TI	Total income, the sum of certain incomes of the statistical unit for the population aged 15 years and over in private households	Economics	Total income (average amount) at the neighborhood level	Toronto Social Development, Finance and Administration	2016	N/A
	HS	Household size, the number of persons in a private household, a characteristic of dwelling		The average number of persons in a household at the neighborhood level		2016	N/A
	PTVM	Percentage of total visible minorities, indirect reflection of genetic diversity; Employment Equity Act defines visible minorities as “persons, other than Aboriginal peoples, who are non-Caucasian in race or non-white in color”	Demographics	Total visible minorities and neighborhood populations		2016	Total visible minority number/neighborhood population

Note: (1) N/A means calculation is not required and detailed values of corresponding indicators have been offered. (2) In the “Calculation” column, all slashes, except for that in “N/A,” mean “divided by”. (3) The letters in the formula of tree diversity represent, respectively, that *n* is the total tree species and *P_i_* means the percentage of species *i* among the total trees at the neighborhood level.
